# Association of Serum Phoenixin-14 and Phoenixin-20 with Diminished Ovarian Reserve

**DOI:** 10.3390/jcm15114356

**Published:** 2026-06-04

**Authors:** Oznur Dundar Akin, Naile Fevziye Misirlioglu, Mete Hakan Karalok, Yeliz Çeçen Dönmez, Gonul Simsek, Hasan Alacam, Hafize Uzun

**Affiliations:** 1 Department of Obstetrics and Gynecology, Faculty of Medicine, Istanbul Atlas University, Istanbul 34408, Türkiye; hakan.karalok@atlas.edu.tr; 2 Department of Biochemistry, Faculty of Medicine, Istanbul Atlas University, Istanbul 34408, Türkiye; nailemisirlioglu@gmail.com (N.F.M.); huzun59@hotmail.com (H.U.); 3 Department of Obstetrics and Gynecology, Kartal Dr. Lütfi Kırdar City Hospital, University of Health Sciences, Istanbul 34668, Türkiye; yelizcecendonmez@gmail.com; 4 Department of Physiology, Faculty of Medicine, Istanbul Atlas University, Istanbul 34408, Türkiye; gonul.simsek@atlas.edu.tr; 5 Department of Medical Biochemistry, Ahenk Laboratory, Istanbul 34381, Türkiye; hasanalacam@hotmail.com

**Keywords:** phoenixin-14, phoenixin-20, ovarian reserve, diminished ovarian reserve, anti-Müllerian hormone, antral follicle count, biomarker, reproductive endocrinology

## Abstract

**Background/Objective:** Phoenixin (PNX), a recently identified neuropeptide, has been shown to regulate the hypothalamic–pituitary–gonadal axis and play a role in folliculogenesis and oocyte maturation. However, its clinical relevance in ovarian reserve remains unclear. This study aimed to evaluate the association between serum PNX-14 and PNX-20 levels and ovarian reserve and to determine whether these peptides provide additional information regarding ovarian reserve status in women with diminished ovarian reserve (DOR). **Methods:** This prospective case–control study included 160 women of reproductive age. Participants were categorized according to anti-Müllerian hormone (AMH) levels and antral follicle count (AFC). Serum PNX-14 and PNX-20 levels were measured using ELISA. Statistical analyses included group comparisons, Spearman correlation, receiver operating characteristic (ROC) analysis, and logistic regression. **Results:** PNX-14 levels differed significantly across AMH-defined groups (*p* < 0.001), with higher levels observed in women with DOR. In contrast, PNX-20 levels showed no significant differences (*p* = 0.305). PNX-14 demonstrated excellent diagnostic performance for identifying DOR (AUC = 0.921), with a sensitivity of 78.7% and a specificity of 95.3% at a cut-off value of 183 pg/mL. A significant negative correlation was found between AMH and PNX-14 (r = −0.639, *p* < 0.001), whereas PNX-20 showed no significant correlation. In logistic regression analysis, PNX-14 was significantly associated with DOR in unadjusted and partially adjusted models; however, this association was attenuated after adjustment for AFC. **Conclusions:** PNX-14 is significantly associated with ovarian reserve status and may provide complementary information regarding DOR when interpreted alongside established ovarian reserve markers. In contrast, PNX-20 does not appear to have clinical utility in this context.

## 1. Introduction

Diminished ovarian reserve (DOR) represents a decline in both the quantity and quality of oocytes and is a major contributor to female infertility. It is commonly assessed using biomarkers such as anti-Müllerian hormone (AMH) and antral follicle count (AFC), which are widely used in clinical practice to estimate ovarian function and reproductive potential [1]. Despite their clinical utility, these markers have limitations in predicting reproductive outcomes, highlighting the need for novel, biologically relevant biomarkers.

Phoenixin (PNX), first identified by Yosten et al. in 2013, is a neuropeptide derived from small integral membrane protein 20 (SMIM20) [2]. It is widely expressed not only in the central nervous system including the hypothalamus, spinal cord, and pituitary gland [2,3] but also in various peripheral tissues such as the heart [3,4], thymus, gastrointestinal tract, spleen, kidneys, lungs [2,5], pancreatic islets [6], adipose tissue [7], ovaries [8,9], and skin [10]. PNX exists mainly in two isoforms, PNX-14 and PNX-20. The amino acid sequence of PNX-14 is highly conserved across multiple species, whereas PNX-20 differs slightly between humans and rodents. Although PNX-20 is predominantly expressed in the hypothalamus and PNX-14 shows higher expression in the heart and spinal cord, both isoforms are believed to exert similar biological functions despite tissue-specific variations [2,10,11,12].

PNX is highly expressed in the hypothalamus and is involved in the modulation of the hypothalamic–pituitary–gonadal (HPG) axis, a key regulator of reproductive function [13]. Experimental studies have demonstrated that PNX enhances gonadotropin-releasing hormone (GnRH) receptor expression, thereby increasing the secretion of luteinizing hormone (LH) and follicle-stimulating hormone (FSH), which are essential for follicular development and ovulation [14].

In addition to its central effects, PNX is also expressed in peripheral reproductive tissues, including the ovaries, suggesting a direct role in folliculogenesis and steroidogenesis. In vitro studies have shown that PNX stimulates granulosa cell proliferation and estradiol production, indicating its involvement in ovarian physiology [14]. Moreover, emerging evidence suggests that PNX may act through the G protein-coupled receptor GPR173, further supporting its regulatory role in reproductive hormone signaling pathways [15].

Recent clinical studies have begun to explore the role of PNX in human reproduction. For example, a prospective study demonstrated that increased serum PNX-14 levels were associated with improved pregnancy outcomes following ovarian stimulation, suggesting a potential role in reproductive success [14]. However, the relationship between PNX and ovarian reserve, particularly in women with DOR, remains poorly understood.

Given the reported involvement of PNX in reproductive hormone regulation and ovarian function, circulating PNX levels may be associated with ovarian reserve status. Therefore, this study aimed to investigate the association between serum PNX-14 and PNX-20 levels and DOR in women and to evaluate whether these peptides provide additional information regarding ovarian reserve status.

## 2. Materials and Methods

### 2.1. Ethical Approval

The study protocol was approved by the Ethics Committee of Istanbul Atlas University (protocol number: E-22686390-050.99-86870; date: 9 December 2025). Written informed consent was obtained from all participants. The study was conducted in accordance with the Declaration of Helsinki.

### 2.2. Study Design and Participants

This prospective case–control study was conducted at the Department of Obstetrics and Gynecology, Atlas University Hospital. A total of 160 women of reproductive age were included and stratified into groups according to ovarian reserve status.

Participants were classified based on serum AMH levels and AFC. DOR was defined as serum AMH < 1.0 ng/mL, which served as the primary study endpoint for receiver operating characteristic (ROC) curve and logistic regression analyses. Participants were further categorized into four groups according to AMH levels: very low (<0.5 ng/mL), decreased (0.5–1.0 ng/mL), normal (1.0–4.0 ng/mL), and high (≥4.0 ng/mL) [16]. AFC was evaluated as a complementary marker of ovarian reserve and was included in multivariable analyses.

Exclusion criteria included age ≥ 40 years, thyroid dysfunction, hyperprolactinemia, and a history of galactorrhea.

### 2.3. Ultrasonographic Evaluation

Transvaginal ultrasonography was performed by the same experienced gynecologist to determine the AFC. AFC was defined as the total number of follicles measuring 2–10 mm in both ovaries.

### 2.4. Sample Collection and Laboratory Analysis

Venous blood samples were collected from all participants following an overnight fast of 10–12 h, between 08:00 and 10:00 a.m. Samples were drawn into serum separator tubes (BD SST II, 5 mL) and EDTA tubes (BD K3 EDTA, 2 mL). After centrifugation at 5000 rpm for 5 min, serum samples were aliquoted and stored at −80 °C until analysis.

Routine hormonal parameters, including AMH, FSH, LH, estradiol (E2), prolactin, and insulin levels, were measured using a Cobas e601 immunoassay analyzer (Roche Diagnostics, Basel, Switzerland).

The homeostasis model assessment of insulin resistance (HOMA-IR) was calculated using the standard formula:

HOMA-IR = (fasting insulin × fasting glucose)/405

### 2.5. Measurement of Serum PNX Levels

Serum PNX-14 and PNX-20 concentrations were measured using commercially available enzyme-linked immunosorbent assay (ELISA) kits (FineTest^®^, Wuhan Fine Biotech Co., Wuhan, China; Catalogue No: EH4521 for PNX-14 and EH4352 for PNX-20). Both assays were based on a sandwich ELISA principle, in which microplates were pre-coated with monoclonal antibodies specific to human PNX isoforms. To minimize matrix effects, serum samples were diluted at least 1:2 with sample dilution buffer before analysis, in accordance with the manufacturer’s recommendations. After incubation with standards and samples, biotin-labeled detection antibodies and HRP-streptavidin conjugates were sequentially applied. The reaction was visualized using tetramethylbenzidine (TMB) substrate, and absorbance was measured at 450 nm using an Epoch™ Microplate Reader (BioTek Instruments, Winooski, VT, USA). The concentration of PNX in each sample was calculated from a standard curve using a four-parameter logistic (4-PL) model. Final PNX-14 and PNX-20 concentrations were calculated by multiplying the measured values by the corresponding dilution factor.

#### 2.5.1. Assay Performance and Precision

The analytical performance of the ELISA kits was defined according to the manufacturer’s specifications. For PNX-20, the detection range was 15.625–1000 pg/mL, with a sensitivity of 9.375 pg/mL. For PNX-14, the detection range was 1.563–100 pg/mL, with a sensitivity of 0.938 pg/mL. The coefficients of variation (CVs) for both intra-assay and inter-assay variability were reported to be less than 10%, indicating acceptable assay reproducibility. All samples were measured in duplicate to minimize analytical variability, and the mean value was used for statistical analyses.

#### 2.5.2. Assay Validation

The analytical performance characteristics of the ELISA kits, including detection range, analytical sensitivity, and intra- and inter-assay precision, were based on the manufacturer’s validation data. No additional in-house validation experiments were performed.

### 2.6. Statistical Analysis

All statistical analyses were performed using IBM SPSS Statistics version 26.0 (IBM Corp., Armonk, NY, USA) and Python version 3.13.1 (Python Software Foundation, Wilmington, DE, USA). The distribution of continuous variables was assessed using the Shapiro–Wilk test. As most variables were not normally distributed, data were expressed as mean ± standard deviation and analyzed using non-parametric tests. Comparisons among more than two groups were performed using the Kruskal–Wallis test. When appropriate, post hoc pairwise comparisons were conducted using the Mann–Whitney U test with Bonferroni correction. Categorical variables were compared using the chi-square test. Correlation analyses between variables were conducted using Spearman’s rank correlation coefficient.

ROC curve and logistic regression analyses were performed using the predefined AMH-based DOR endpoint. Because participants were categorized according to AMH levels, these analyses were intended to assess the discriminatory capacity of PNX isoforms relative to an established ovarian reserve marker rather than to validate them against an independent clinical outcome. ROC curve analysis was performed to evaluate the diagnostic performance of PNX-14 and PNX-20 in distinguishing DOR (AMH < 1 ng/mL). The area under the curve (AUC), sensitivity, specificity, and optimal cut-off values were calculated using the Youden index.

Binary logistic regression analysis was used to assess the association between PNX-14 and DOR. Both univariate and multivariate models were constructed. In multivariate analysis, potential confounding variables including age, FSH, and antral follicle count (AFC) were included. Results were reported as β coefficients, odds ratios (ORs), and 95% confidence intervals (CIs). Model calibration was assessed using the Hosmer–Lemeshow goodness-of-fit test. A two-tailed *p*-value < 0.05 was considered statistically significant.

## 3. Results

A total of 160 women were stratified into four groups according to AMH levels: very low (<0.5 ng/mL), decreased (0.5–1.0 ng/mL), normal (1.0–4.0 ng/mL), and high (≥4.0 ng/mL). As shown in Table 1, there were significant differences among groups in terms of age, AMH, AFC, FSH, LH, and E2 levels (all *p* < 0.001). Women with lower AMH levels exhibited significantly higher age, FSH, and LH levels, along with lower AFC values compared to those with normal and high ovarian reserve. Estradiol levels were also significantly different across groups. In contrast, no significant differences were observed in BMI, prolactin, glucose, insulin, and HOMA-IR levels among the groups (*p* > 0.05 for all), indicating comparable metabolic and endocrine profiles independent of ovarian reserve status. These findings suggest that AMH-based ovarian reserve classification is strongly associated with reproductive hormonal parameters but not with metabolic indicators.

Serum PNX levels across AMH-defined ovarian reserve categories are presented in Table 2. PNX-14 levels differed significantly among the groups (*p* < 0.001), with the highest concentrations observed in women with decreased ovarian reserve (0.5–1.0 ng/mL) and very low AMH levels (<0.5 ng/mL), followed by lower levels in the normal and high AMH groups. This pattern indicates a progressive decrease in PNX-14 concentrations with increasing ovarian reserve. In contrast, serum PNX-20 levels did not differ significantly among the AMH categories (*p* = 0.305), despite a numerical trend toward higher levels in the very low AMH group. These findings suggest that PNX-14, but not PNX-20, is significantly associated with ovarian reserve status and may provide complementary information regarding DOR.

### 3.1. Receiver Operating Characteristic (ROC) Analysis

**Figure 1:**
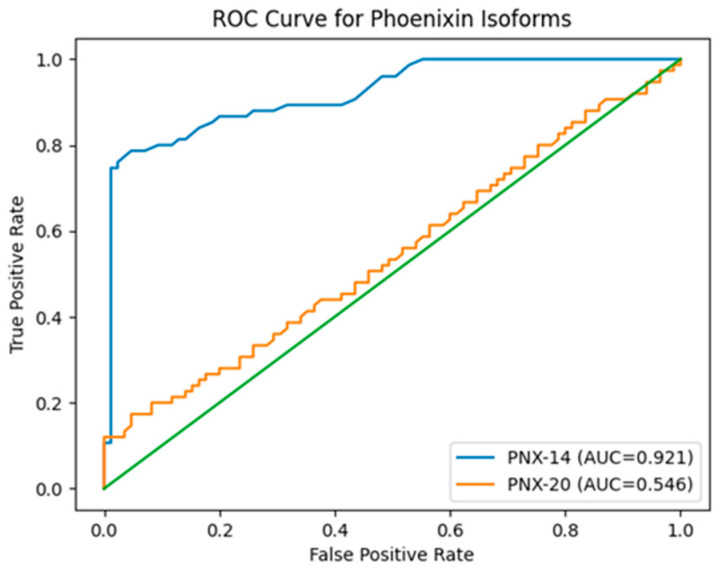
Receiver operating characteristic (ROC) curves of phoenixin-14 and phoenixin-20 for the detection of diminished ovarian reserve (AMH < 1 ng/mL).

ROC curve analysis was performed to evaluate the diagnostic performance of PNX isoforms in distinguishing women with DOR (AMH < 1 ng/mL) from those with normal ovarian reserve. PNX-14 demonstrated excellent diagnostic accuracy, with an area under the curve (AUC) of 0.921. The optimal cut-off value was identified as 183 pg/mL, yielding a sensitivity of 78.7% and a specificity of 95.3%, indicating high diagnostic accuracy. In contrast, PNX-20 showed poor diagnostic performance, with an AUC of 0.546, indicating no significant discriminative ability. Although specificity remained high, the sensitivity was markedly low (17.3%), limiting its clinical utility (Table 3, Figure 1).

**Figure 2:**
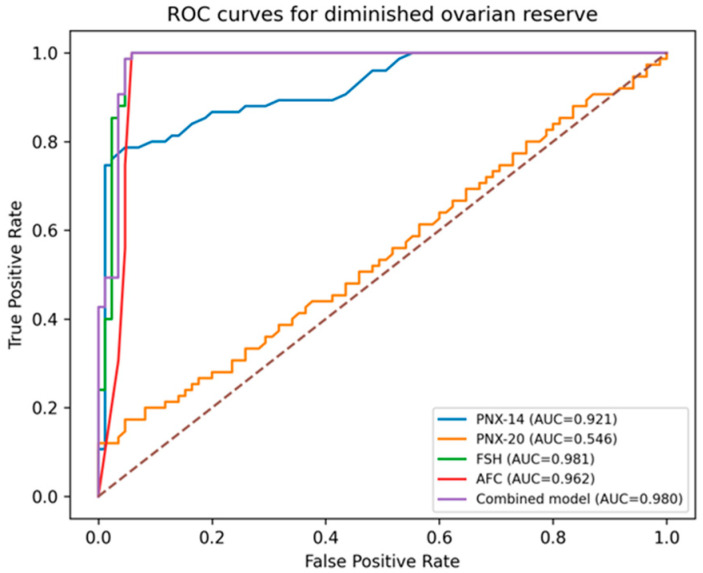
ROC curves for diminished ovarian reserve.

In comparison, PNX-20 showed poor diagnostic accuracy (AUC = 0.546). Among conventional markers, FSH and AFC also demonstrated good discriminative ability, while the combined model (PNX-14, age, FSH, and AFC) provided the highest diagnostic performance. ROC curves for PNX-14, PNX-20, FSH, AFC, and the combined model in detecting DOR (AMH < 1 ng/mL) (Table 4, Figure 2).

#### 3.1.1. Logistic Regression Analysis

Binary logistic regression analysis demonstrated that PNX-14 was independently associated with DOR (AMH < 1 ng/mL) (β = 0.071, *p* < 0.001). These findings indicate that higher PNX-14 levels are associated with an increased likelihood of DOR. However, the association was attenuated after adjustment for AFC, suggesting that PNX-14 may serve as an adjunctive rather than independent marker of ovarian reserve (Table 5). In logistic regression analysis, PNX-14 was significantly associated with DOR in the unadjusted model (OR = 1.073, *p* < 0.001) and remained significant after adjustment for age and FSH (*p* = 0.001). However, when AFC was included in the fully adjusted model, the association weakened and did not reach statistical significance (*p* = 0.071). These findings suggest that PNX-14 is strongly associated with ovarian reserve and demonstrates high diagnostic performance, although its independence may be influenced by established markers such as AFC.

#### 3.1.2. Correlation Analysis

Spearman correlation analysis revealed a significant negative correlation between AMH and PNX-14 levels (r = −0.639, *p* < 0.001), indicating that higher PNX-14 levels are associated with lower ovarian reserve. In contrast, no significant correlation was observed between AMH and PNX-20 levels (r = −0.132, *p* = 0.097) (Table 6).

## 4. Discussion

The present study demonstrates that serum PNX-14 levels are significantly associated with ovarian reserve status in women and exhibit strong diagnostic performance in identifying *DOR*. PNX-14 concentrations were significantly elevated in women with reduced ovarian reserve and showed a robust negative correlation with AMH levels. Moreover, ROC analysis revealed excellent discriminative ability of PNX-14, whereas PNX-20 showed no significant association or diagnostic utility. Although PNX-14 remained significantly associated with DOR in unadjusted and partially adjusted models, this association was attenuated after adjustment for AFC, suggesting that its predictive value may be influenced by established ovarian reserve markers.

Women with lower AMH levels demonstrated significantly reduced AFC together with increased FSH concentrations, which is consistent with the established endocrine profile of DOR. Previous studies have shown that AMH and AFC are strongly correlated markers reflecting the remaining follicular pool, whereas elevated FSH levels are considered a compensatory response to declining ovarian function [17,18,19,20,21]. In our cohort, age progressively increased as AMH levels decreased, further supporting the well-recognized age-related decline in ovarian reserve. In contrast, BMI, glucose metabolism parameters, prolactin, and HOMA-IR values did not differ significantly among groups, suggesting that the observed differences in PNX-14 levels were more closely associated with ovarian reserve status rather than metabolic disturbances.

The association between PNX and female reproductive disorders has recently attracted increasing attention, particularly in conditions characterized by neuroendocrine dysregulation such as polycystic ovary syndrome (PCOS). Previous studies have demonstrated altered PNX expression both centrally and peripherally in experimental and clinical models of PCOS [8,22,23]. Kalamon et al. [8] reported increased PNX-14 and GPR173 expression in the hypothalamus, ovary, and periovarian adipose tissue in a rat model of PCOS, suggesting a potential role of the PNX system in ovarian dysfunction and altered reproductive signaling. Similarly, Ullah et al. [22] demonstrated elevated circulating PNX-14 levels in women with PCOS, which were positively associated with luteinizing hormone concentrations, further supporting the interaction between PNX and the hypothalamic–pituitary–gonadal axis. Although PCOS and DOR represent distinct reproductive phenotypes, both conditions involve dysregulation of ovarian endocrine function and follicular dynamics. Although elevated PNX-14 levels were observed in women with DOR, the mechanistic basis of this association remains speculative and warrants further investigation. The absence of significant changes in PNX-20 further suggests that different PNX isoforms may exert distinct biological roles in reproductive regulation.

ROC analysis demonstrated that PNX-14 had excellent diagnostic performance for identifying DOR, with an AUC of 0.921 and high specificity. In contrast, PNX-20 showed poor discriminative ability and markedly low sensitivity, limiting its clinical applicability. Interestingly, although PNX-14 exhibited strong diagnostic performance, conventional ovarian reserve markers such as FSH and AFC demonstrated even higher AUC values in our cohort. Moreover, the combined model including PNX-14, age, FSH, and AFC achieved the highest overall diagnostic accuracy, suggesting that PNX-14 may provide additional supportive information when interpreted together with established ovarian reserve parameters rather than functioning as a standalone marker. Since ovarian reserve assessment currently relies on a combination of biochemical and ultrasonographic parameters rather than a single biomarker, PNX-14 may serve as a complementary biomarker alongside established ovarian reserve markers; however, its clinical utility requires further validation [2,8,17,18,19,20,21,22,23].

Previous studies have demonstrated that PNX-14 may regulate ovarian cyclicity through modulation of the hypothalamic–pituitary–gonadal axis [24]. PNX-14 has been shown to enhance GnRH receptor expression in pituitary gonadotrophs, thereby increasing pituitary sensitivity to GnRH stimulation and contributing to the preovulatory LH surge [2]. Interestingly, PNX-14 does not appear to directly stimulate LH secretion itself, suggesting that its primary role may involve sensitization of the pituitary to GnRH action rather than direct endocrine stimulation [25]. In this context, the reduced/altered PNX-14 levels observed in women with DOR in the present study may reflect impaired neuroendocrine regulation of ovarian function. These findings support an association between PNX-14 and ovarian reserve status. Although previous studies have linked PNX signaling to reproductive function, the biological mechanisms underlying this relationship remain unclear and warrant further investigation [14].

Spearman correlation analysis demonstrated significant negative correlations between PNX-14 and both AMH and AFC, while positive correlations were observed with FSH, LH, and age. These findings suggest that elevated PNX-14 levels are associated with DOR and ovarian aging. In contrast, no significant correlation was identified between AMH and PNX-20, supporting the possibility that PNX isoforms may differ in their biological relevance to ovarian physiology. Logistic regression analysis further confirmed the association between PNX-14 and DOR. Although PNX-14 remained significant after adjustment for age and FSH, this association lost significance after inclusion of AFC in the fully adjusted model. Since AFC is a well-established indicator of the remaining follicular pool, these findings suggest that the predictive value of PNX-14 may partially overlap with conventional ovarian reserve markers. Nevertheless, the strong correlations and diagnostic performance of PNX-14 suggest that it may provide complementary information regarding ovarian reserve status. Consistent with previous reports linking PNX-14 to reproductive outcomes, our findings demonstrate significant associations between PNX-14 and established ovarian reserve markers. However, the underlying biological mechanisms remain unclear and require further investigation [14].

### 4.1. Strengths

This study has several strengths. First, it is one of the few clinical studies investigating the relationship between circulating PNX isoforms and ovarian reserve in women, thereby contributing novel data to the existing literature. Second, the prospective case–control design and well-defined inclusion criteria enhanced the internal validity of the findings. Third, both PNX-14 and PNX-20 were evaluated simultaneously, allowing a direct comparison between isoforms and demonstrating the differential clinical relevance of PNX-14. In addition, ovarian reserve was assessed using established and widely accepted markers, including AMH and AFC, which strengthens the clinical applicability of the results. The use of standardized ELISA kits and duplicate measurements further improved the reliability and reproducibility of the biochemical analyses. Finally, multiple statistical approaches, including group comparisons, correlation analysis, ROC curve analysis, and logistic regression, were applied, providing a comprehensive evaluation of the association between PNX levels and ovarian reserve.

### 4.2. Limitations

Several limitations of this study should be acknowledged. First, although the sample size was sufficient to achieve adequate statistical power, the relatively modest cohort and single-center design may limit the generalizability of the findings. Second, the cross-sectional nature of the study precludes any conclusions regarding causality between circulating PNX levels and ovarian reserve status. Third, although PNX-14 demonstrated strong diagnostic performance, its association with DOR was attenuated after adjustment for AFC, suggesting that its predictive value may partially overlap with established ovarian reserve markers. Fourth, potential confounding factors, including lifestyle characteristics and unmeasured hormonal or metabolic variables, were not comprehensively evaluated and may have influenced the observed associations. In addition, several potentially relevant reproductive and clinical factors, including menstrual cycle phase, smoking status, endometriosis, previous ovarian surgery, contraceptive use, infertility-related characteristics, and polycystic ovary syndrome, were not systematically recorded and therefore could not be included in the multivariable analyses. Consequently, residual confounding cannot be excluded. Furthermore, ovarian reserve categories and the primary diagnostic endpoint were based on AMH levels. Therefore, the diagnostic performance of PNX-14 was assessed against an AMH-defined outcome rather than an independent reproductive endpoint, which should be considered when interpreting the findings. Finally, the underlying molecular mechanisms linking PNX to ovarian physiology were not investigated. Further experimental studies are needed to elucidate the role of PNX in folliculogenesis, ovarian aging, and reproductive endocrinology.

### 4.3. Future Directions

Future studies should validate the association between PNX-14 and ovarian reserve in larger, multicenter cohorts and explore its predictive value for reproductive outcomes in longitudinal designs. Combining PNX-14 with established markers such as AMH, AFC, and FSH may improve diagnostic accuracy, although external validation is required. Further experimental research is needed to clarify the molecular mechanisms of PNX in ovarian physiology, particularly its role in the hypothalamic–pituitary–gonadal axis and granulosa cell function. Additionally, investigating its potential role in other reproductive disorders may expand its clinical relevance.

## 5. Conclusions

In conclusion, serum PNX-14 levels are significantly associated with ovarian reserve status in women and demonstrate strong diagnostic performance in identifying DOR. In contrast, PNX-20 does not appear to have clinical relevance in this context. Although the association between PNX-14 and DOR was attenuated after adjustment for AFC, the overall findings suggest that PNX-14 may provide complementary information when interpreted alongside established ovarian reserve markers such as AMH and AFC. Further large-scale prospective and mechanistic studies are warranted to confirm these findings and clarify the role of PNX-14 in reproductive physiology.

## Figures and Tables

**Table 1:** Clinical and hormonal characteristics according to AMH categories.

Parameter	Very Low (<0.5)	Decreased (0.5–1.0)	Normal (1.0–4.0)	High (≥4.0)	*p*-Value
**Age (Year)**	33.59 ± 5.47	31.84 ± 5.22	28.17 ± 5.39	27.19 ± 4.46	<0.001
**BMI (kg/m^2^)**	24.69 ± 2.90	25.83 ± 2.95	25.45 ± 2.65	24.86 ± 3.00	0.296
**AMH (ng/mL)**	0.19 ± 0.14	0.75 ± 0.14	2.73 ± 0.83	5.32 ± 0.89	<0.001
**AFC (count)**	3.46 ± 1.17	4.95 ± 1.20	9.58 ± 2.10	13.74 ± 2.63	<0.001
**FSH (IU/L)**	13.19 ± 2.86	11.52 ± 2.64	8.41 ± 2.12	6.46 ± 1.56	<0.001
**LH (IU/L)**	8.06 ± 2.21	7.58 ± 2.18	6.25 ± 1.88	4.98 ± 1.58	<0.001
**E2 (pg/mL)**	66.13 ± 16.93	64.42 ± 16.07	81.82 ± 22.47	90.45 ± 22.69	<0.001
**Prolactin (ng/mL)**	13.75 ± 5.62	12.41 ± 5.11	12.94 ± 5.33	13.85 ± 5.49	0.380
**Glucose mg/dL**	91.90 ± 5.61	90.32 ± 5.48	89.74 ± 5.22	91.47 ± 5.35	0.199
**Insulin (µIU/mL)**	9.46 ± 2.57	8.72 ± 2.44	9.84 ± 2.63	11.09 ± 2.30	0.078
**HOMA-IR**	2.14 ± 0.57	1.94 ± 0.53	2.16 ± 0.58	2.50 ± 0.54	0.086

Data are presented as mean ± standard deviation. *p*-values were calculated using the Kruskal–Wallis test. A *p*-value < 0.05 was considered statistically significant. **BMI**, body mass index; **AMH**, anti-Müllerian hormone; **AFC**, antral follicle count; **FSH**, Follicle-stimulating hormone; **LH**, luteinizing hormone; **E2**, estradiol; **HOMA-IR**, homeostasis model assessment of insulin resistance.

**Table 2:** Serum phoenixin levels according to AMH categories.

Parameter	Very Low (<0.5)	Decreased (0.5–1.0)	Normal (1.0–4.0)	High (≥4.0)	*p*-Value
**Phoenixin-14 (pg/mL)**	208.03 ± 36.39	226.61 ± 37.75	156.52 ± 23.61	149.85 ± 20.43	<0.001
**Phoenixin-20 (pg/mL)**	476.35 ± 173.96	443.08 ± 191.21	447.16 ± 163.64	392.04 ± 154.64	0.305

Data are presented as mean ± standard deviation. *p*-values were calculated using the Kruskal–Wallis test.

**Table 3:** Diagnostic performance of phoenixin isoforms.

Marker	AUC	Cut-Off	Sensitivity	Specificity
**Phoenixin-14**	0.921	183 pg/mL	78.7%	95.3%
**Phoenixin-20**	0.546	673 pg/mL	17.3%	95.3%

Cut-off values were determined using the Youden index.

**Table 4:** Diagnostic performance of PNX isoforms and conventional ovarian reserve markers.

Marker/Model	AUC	Cut-Off	Sensitivity	Specificity
**PNX-14**	0.921	183 pg/mL	78.7%	95.3%
**PNX-20**	0.546	673 pg/mL	17.3%	95.3%
**FSH**	0.981	9.08 IU/L	100.0%	94.1%
**AFC**	0.962	≤5 follicles	100.0%	94.1%
**Combined model**	0.980	0.322 probability	100.0%	94.1%

Cut-off values were determined using the Youden index. For AFC, lower values indicate diminished ovarian reserve; therefore, the diagnostic threshold is presented as ≤5 follicles.

**Table 5:** Logistic regression analysis for diminished ovarian reserve.

Model	Variable	β	Odds Ratio (OR)	95% CI for OR	*p*-Value
**Unadjusted model**	PNX14	0.071	1.073	1.049–1.099	<0.001
**Adjusted model 1**	PNX14	0.071	1.074	1.047–1.101	<0.001
**Adjusted model 1**	Age	0.123	1.131	1.034–1.238	0.007
**Adjusted model 2**	PNX14	0.044	1.045	1.017–1.074	0.001
**Adjusted model 2**	Age	−0.010	0.990	0.867–1.131	0.885
**Adjusted model 2**	FSH	0.799	2.223	1.576–3.135	<0.001
**Fully adjusted model**	PNX14	0.026	1.026	0.998–1.055	0.071
**Fully adjusted model**	Age	−0.013	0.987	0.854–1.141	0.860
**Fully adjusted model**	FSH	0.420	1.522	1.040–2.226	0.031
**Fully adjusted model**	AFC	−0.425	0.654	0.443–0.965	0.033

Dependent variable: diminished ovarian reserve (AMH < 1 ng/mL). Adjusted model 1 includes PNX-14 and age; adjusted model 2 includes PNX-14, age, and FSH; fully adjusted model includes PNX-14, age, FSH, and AFC. **β**, regression coefficient; **OR**, odds ratio; **CI**, confidence interval.

**Table 6:** Spearman correlation analysis between PNX isoforms and ovarian reserve parameters.

Marker	Variable	Spearman (r)	*p*-Value
**PNX-14**	AMH	−0.639	<0.001
**PNX-14**	AFC	−0.661	<0.001
**PNX-14**	FSH	0.638	<0.001
**PNX-14**	LH	0.364	<0.001
**PNX-14**	E2	−0.393	<0.001
**PNX-14**	Age	0.390	<0.001

**AMH**, anti-Müllerian hormone; **AFC**, antral follicle count; **FSH**, follicle-stimulating hormone; **LH**, luteinizing hormone; **E2**, estradiol; **PNX**, phoenixin.

## Data Availability

The datasets generated and/or analyzed in the current study are available from the corresponding author upon reasonable request. All data supporting the findings of this study are included within the article.

## References

[1] Tas M., Oner G., Ulug P., Yavuz A., Ozcelik B. (2019). Evaluation of protective effects of GnRH agonist or antagonist on ovarian reserve with anti-Müllerian hormone and histological analysis in a rat model using cisplatin. Arch. Med. Sci..

[2] Yosten G.L., Lyu R.M., Hsueh A.J., Avsian-Kretchmer O., Chang J., Tullock C.W., Dun S.L., Dun N., Samson W.K. (2013). A novel reproductive peptide, phoenixin. J. Neuroendocrinol..

[3] Lyu R.M., Huang X.F., Zhang Y., Dun S.L., Luo J.J., Chang J.K., Dun N.J. (2013). Phoenixin: A novel peptide in rodent sensory ganglia. Neuroscience.

[4] Rocca C., Scavello F., Granieri M.C., Pasqua T., Amodio N., Imbrogno S., Gattuso A., Mazza R., Cerra M.C., Angelone T. (2018). Phoenixin-14: Detection and novel physiological implications in cardiac modulation and cardioprotection. Cell. Mol. Life Sci..

[5] Prinz P., Scharner S., Friedrich T., Schalla M., Goebel-Stengel M., Rose M., Stengel A. (2017). Central and peripheral expression sites of phoenixin-14 immunoreactivity in rats. Biochim. Biophys. Res. Commun..

[6] Billert M., Kołodziejski P.A., Strowski M.Z., Nowak K.W., Skrzypski M. (2019). Phoenixin-14 stimulates proliferation and insulin secretion in insulin producing INS-1E cells. Biochim. Biophys. Acta Mol. Cell Res..

[7] Billert M., Wojciechowicz T., Jasaszwili M., Szczepankiewicz D., Waśko J., Kaźmierczak S., Strowski M.Z., Nowak K.W., Skrzypski M. (2018). Phoenixin-14 stimulates differentiation of 3T3-L1 preadipocytes via cAMP/Epac-dependent mechanism. Biochim. Biophys. Acta Mol. Cell Biol. Lipids.

[8] Kalamon N., Błaszczyk K., Szlaga A., Billert M., Skrzypski M., Pawlicki P., Wójtowicz E.G., Balak M.K., Blasiak A., Rak A. (2020). Levels of the neuropeptide phoenixin-14 and its receptor GRP173 in the hypothalamus, ovary and periovarian adipose tissue in rat model of polycystic ovary syndrome. Biochem. Biophys. Res. Commun..

[9] Nguyen X.P., Nakamura T., Osuka S., Bayasula B., Nakanishi N., Kasahara Y., Muraoka A., Hayashi S., Nagai T., Murase T. (2019). Effect of the neuropeptide phoenixin and its receptor GPR173 during folliculogenesis. Reproduction.

[10] Cowan A., Lyu R.M., Chen Y.H., Dun S.L., Chang J.K., Dun N.J. (2015). Phoenixin: A candidate pruritogen in the mouse. Neuroscience.

[11] Stein L.M., Haddock C.J., Samson W.K., Kolar G.R., Yosten G.L.C. (2018). The phoenixins: From discovery of the hormone to identification of the receptor and potential physiologic actions. Peptides.

[12] Mcilwraith E.K., Belsham D.D. (2018). Phoenixin: Uncovering its receptor, signaling and functions. Acta Pharmacol. Sin..

[13] Stein L.M., Tullock C.W., Mathews S.K., Garcia-Galiano D., Elias C.F., Samson W.K., Yosten G.L.C. (2016). Hypothalamic action of phoenixin to control reproductive hormone secretion in females: Importance of the orphan G protein-coupled receptor Gpr173. Am. J. Physiol. Regul. Integr. Comp. Physiol..

[14] Piróg M., Jach R., Ząbczyk M., Natorska J. (2023). Increased Serum Levels of Phoenixin-14, Nesfatin-1 and Dopamine Are Associated with Positive Pregnancy Rate after Ovarian Stimulation. J. Clin. Med..

[15] Muzammil A.N., Barathan M., Yazid M.D., Sulaiman N., Makpol S., Ibrahim N.M., Jaafar F., Abdullah N.A.H. (2024). A systematic scoping review of the multifaceted role of phoenixin in metabolism: Insights from in vitro and in vivo studies. Front. Endocrinol..

[16] Committee on Gynecologic Practice (2015). Committee opinion no. 618: Ovarian reserve testing. Obstet. Gynecol..

[17] Broer S.L., Broekmans F.J., Laven J.S., Fauser B.C. (2014). Anti-Müllerian hormone: Ovarian reserve testing and its potential clinical implications. Hum. Reprod. Update.

[18] La Marca A., Sunkara S.K. (2014). Individualization of controlled ovarian stimulation in IVF using ovarian reserve markers: From theory to practice. Hum. Reprod. Update.

[19] Dewailly D., Andersen C.Y., Balen A., Broekmans F., Dilaver N., Fanchin R., Griesinger G., Kelsey T.W., La Marca A., Lambalk C. (2014). The physiology and clinical utility of anti-Mullerian hormone in women. Hum. Reprod. Update.

[20] Steiner A.Z., Herring A.H., Kesner J.S., Meadows J.W., Stanczyk F.Z., Hoberman S., Baird D.D. (2011). Antimüllerian hormone as a predictor of natural fecundability in women aged 30–42 years. Obstet. Gynecol..

[21] Shahrokh Tehraninezhad E., Mehrabi F., Taati R., Kalantar V., Aziminekoo E., Tarafdari A. (2016). Analysis of ovarian reserve markers (AMH, FSH, AFC) in different age strata in IVF/ICSI patients. Int. J. Reprod. Biomed..

[22] Ullah K., Ur Rahman T., Wu D.D., Lin X.-H., Liu Y., Guo X.-Y., Leung P.C., Zhang R.-J., Huang H.-F., Sheng J.-Z. (2017). Phoenixin-14 concentrations are increased in association with luteinizing hormone and nesfatin-1 concentrations in women with polycystic ovary syndrome. Clin. Chim. Acta.

[23] Szeliga A., Rudnicka E., Maciejewska-Jeske M., Kucharski M., Kostrzak A., Hajbos M., Niwczyk O., Smolarczyk R., Meczekalski B. (2022). Neuroendocrine Determinants of Polycystic Ovary Syndrome. Int. J. Environ. Res. Public Health.

[24] Pałasz A., Rojczyk E., Bogus K., Worthington J.J., Wiaderkiewicz R. (2015). The novel neuropeptide phoenixin is highly co-expressed with nesfatin-1 in the rat hypothalamus, an immunohistochemical study. Neurosci. Lett..

[25] Friedrich T., Stengel A. (2021). Role of the Novel Peptide Phoenixin in Stress Response and Possible Interactions with Nesfatin-1. Int. J. Mol. Sci..

